# Supplementary material for: The METLIN small molecule dataset for machine learning-based retention time prediction
Source: Nat Commun. 2019 Dec 20;10:5811. doi: 10.1038/s41467-019-13680-7 (PMC6925099; doi:10.1038/s41467-019-13680-7)
Supplement: Supplementary file 1 — Supplementary Information [file 41467_2019_13680_MOESM1_ESM.pdf]

Supplementary Information

The METLIN small molecule retention time dataset for machine learning-based prediction

Domingo-Almenara et al.

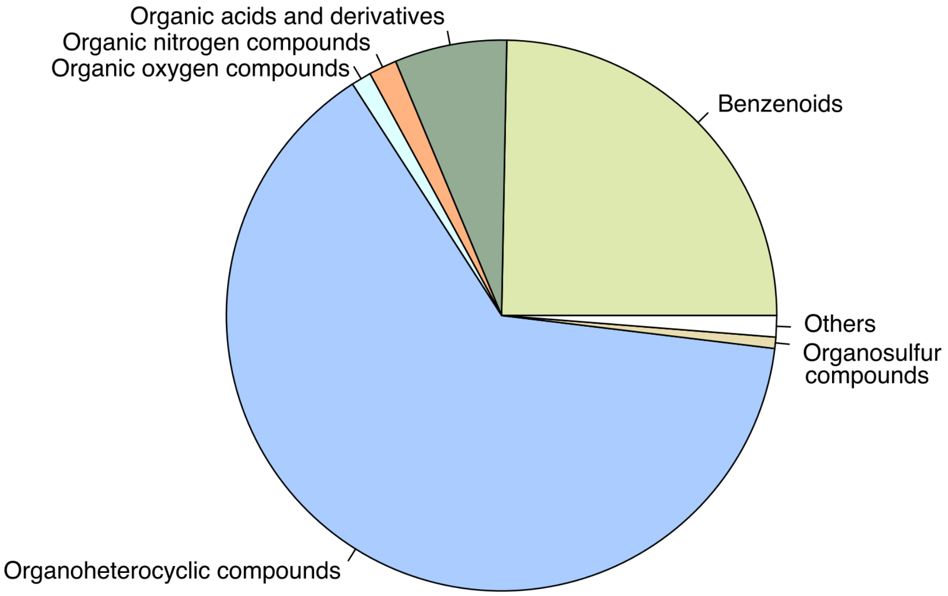

Supplementary Figure 1. The SMRT dataset molecules chemical classification by ClassyFire (superclass level).

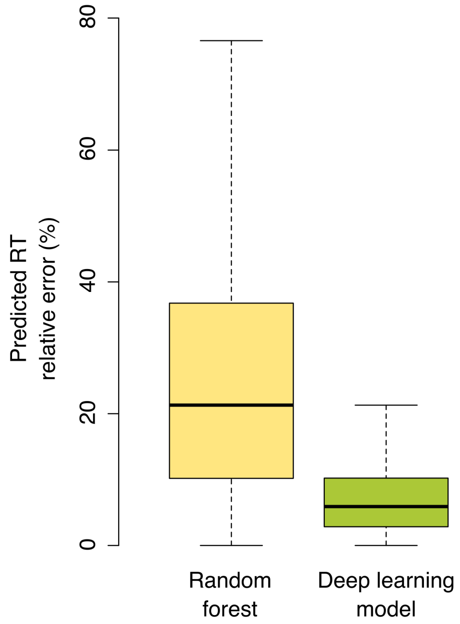

Supplementary Figure 2. RT prediction performance comparison between a random forest and a deep learning model.

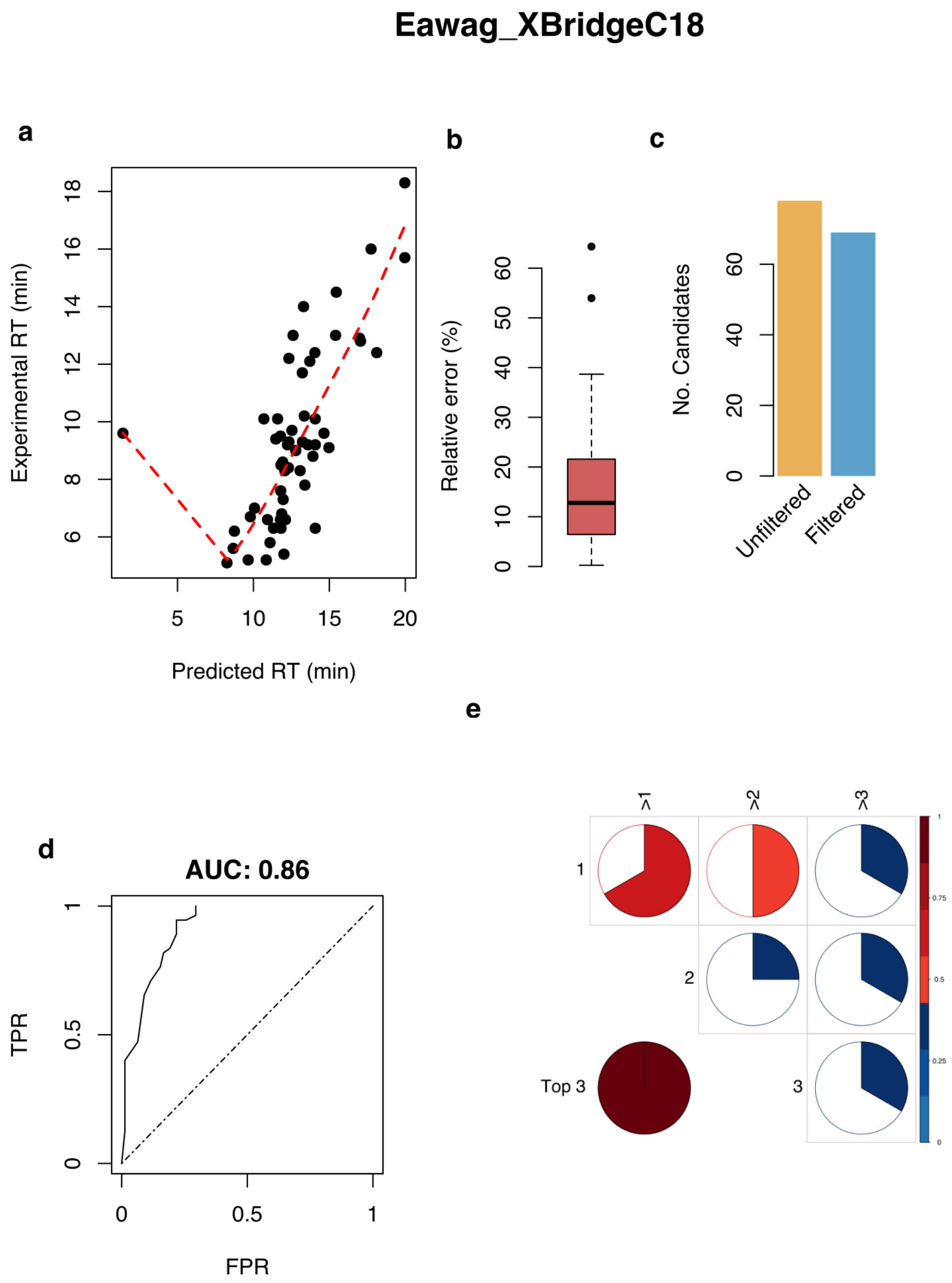

Supplementary Figure 3. Predicted-experimental projections results. Panels **a** shows the projection between the experimental and predicted RT. Panel **b** shows relative errors between the experimental and predicted RT. Box plots represent median value and their error bars represent the interquartile range (25%-75% percentiles). Panel **c** shows the number of putative candidates before and after filtering by RT error threshold. Panels **d** shows the receiver operating characteristic (ROC) curves with their respective area under curve (AUC) values. Panels **e** shows the candidate ranking. Ranking panels show the percentage of molecules in which the correct identity is exactly the first, second or third top candidate (y-axis, correct candidate ranking) and there are more than 1, 2 or 3 putative candidates in total (x-axis, total number of candidates). The *top 3* cell shows the percentage of cases in which the correct identity was ranked among the top 3 candidates of the total of cases with more than 3 candidates.

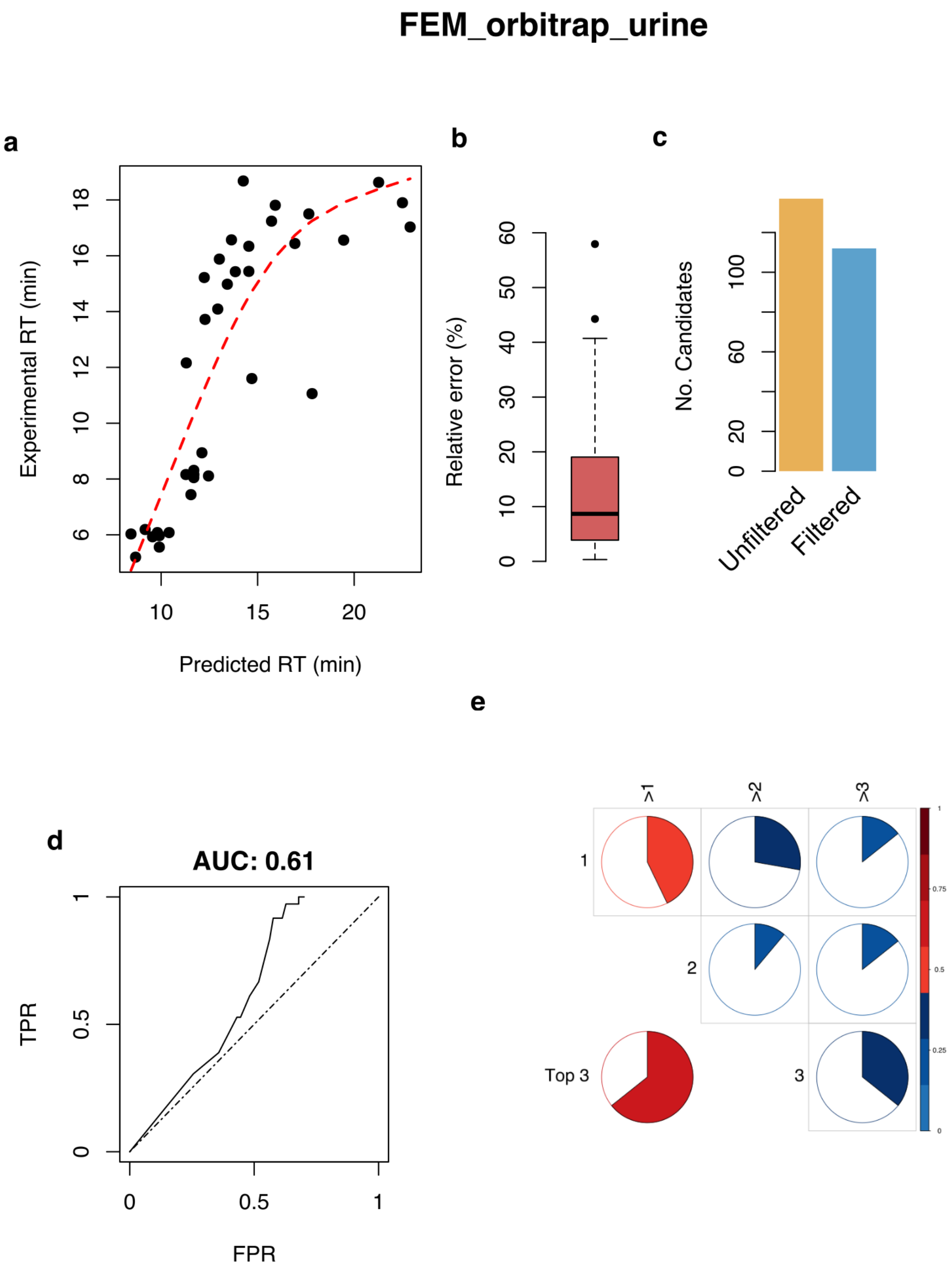

Supplementary Figure 4. Predicted-experimental projections results. Panels **a** shows the projection between the experimental and predicted RT. Panel **b** shows relative errors between the experimental and predicted RT. Box plots represent median value and their error bars represent the interquartile range (25%-75% percentiles). Panel **c** shows the number of putative candidates before and after filtering by RT error threshold. Panels **d** shows the receiver operating characteristic (ROC) curves with their respective area under curve (AUC) values. Panels **e** shows the candidate ranking. Ranking panels show the percentage of molecules in which the correct identity is exactly the first, second or third top candidate (y-axis, correct candidate ranking) and there are more than 1, 2 or 3 putative candidates in total (x-axis, total number of candidates). The *top 3* cell shows the percentage of cases in which the correct identity was ranked among the top 3 candidates of the total of cases with more than 3 candidates.

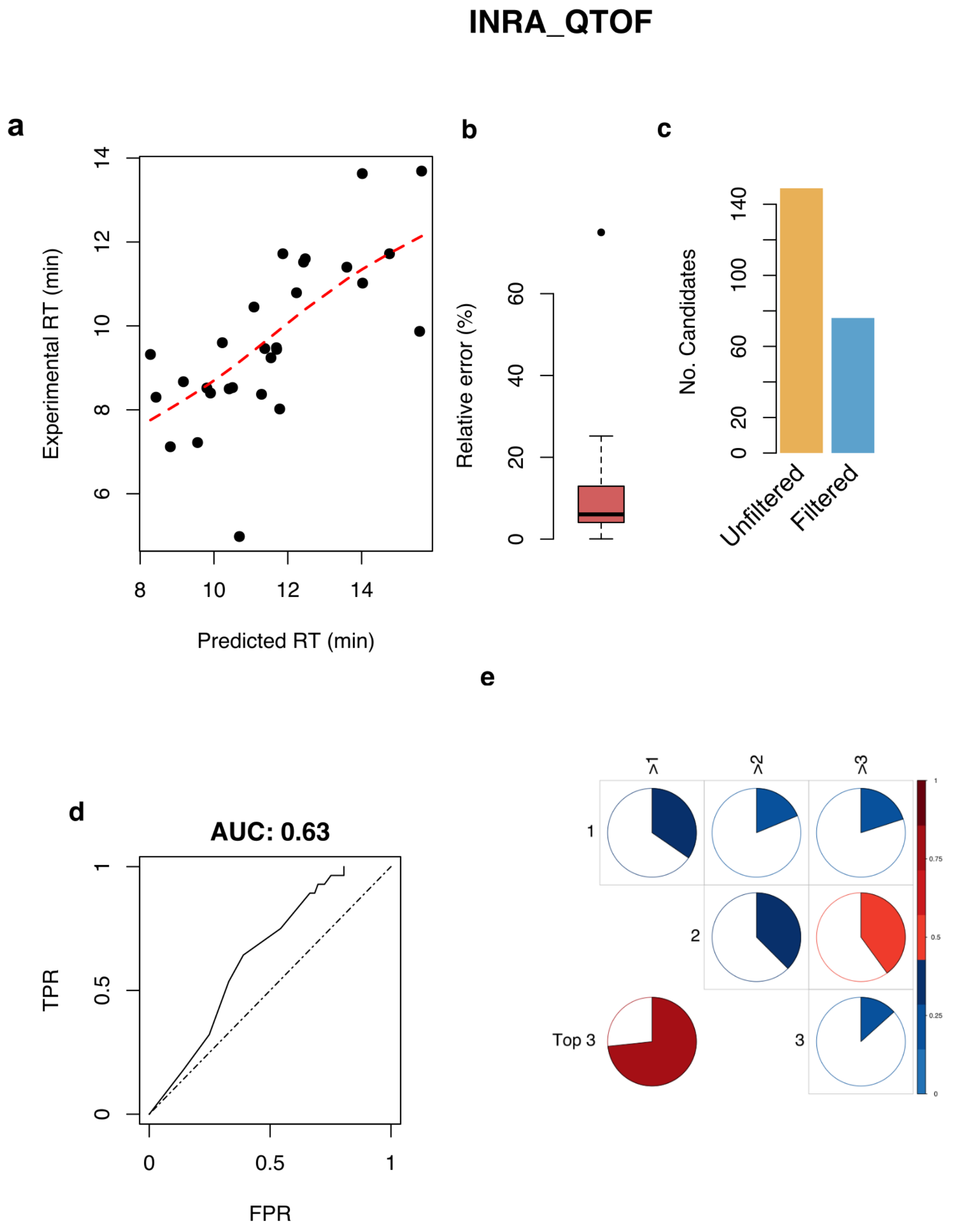

Supplementary Figure 5. Predicted-experimental projections results. Panels **a** shows the projection between the experimental and predicted RT. Panel **b** shows relative errors between the experimental and predicted RT. Box plots represent median value and their error bars represent the interquartile range (25%-75% percentiles). Panel **c** shows the number of putative candidates before and after filtering by RT error threshold. Panels **d** shows the receiver operating characteristic (ROC) curves with their respective area under curve (AUC) values. Panels **e** shows the candidate ranking. Ranking panels show the percentage of molecules in which the correct identity is exactly the first, second or third top candidate (y-axis, correct candidate ranking) and there are more than 1, 2 or 3 putative candidates in total (x-axis, total number of candidates). The *top 3* cell shows the percentage of cases in which the correct identity was ranked among the top 3 candidates of the total of cases with more than 3 candidates.

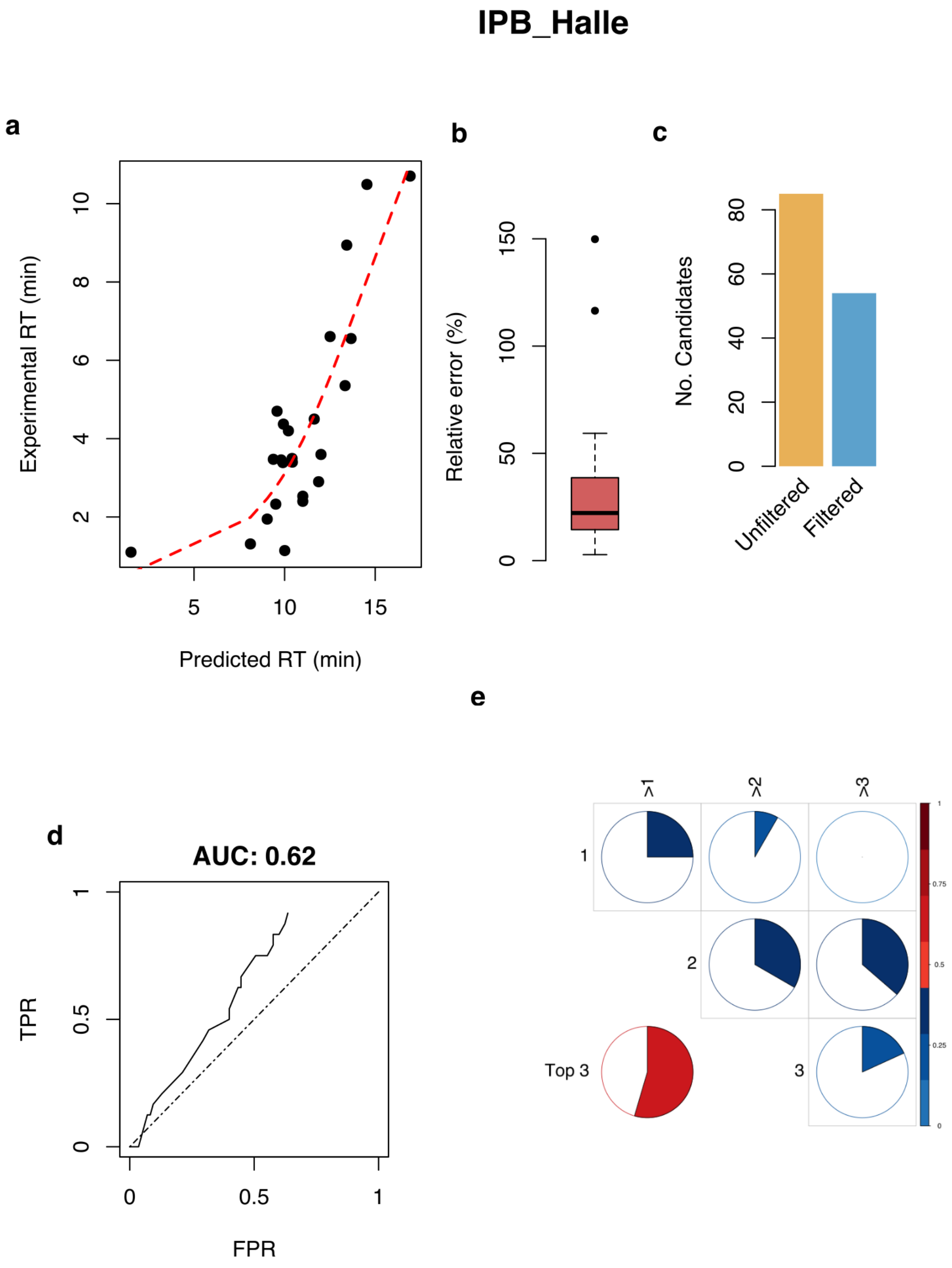

Supplementary Figure 6. Predicted-experimental projections results. Panels **a** shows the projection between the experimental and predicted RT. Panel **b** shows relative errors between the experimental and predicted RT. Box plots represent median value and their error bars represent the interquartile range (25%-75% percentiles). Panel **c** shows the number of putative candidates before and after filtering by RT error threshold. Panels **d** shows the receiver operating characteristic (ROC) curves with their respective area under curve (AUC) values. Panels **e** shows the candidate ranking. Ranking panels show the percentage of molecules in which the correct identity is exactly the first, second or third top candidate (y-axis, correct candidate ranking) and there are more than 1, 2 or 3 putative candidates in total (x-axis, total number of candidates). The *top 3* cell shows the percentage of cases in which the correct identity was ranked among the top 3 candidates of the total of cases with more than 3 candidates.

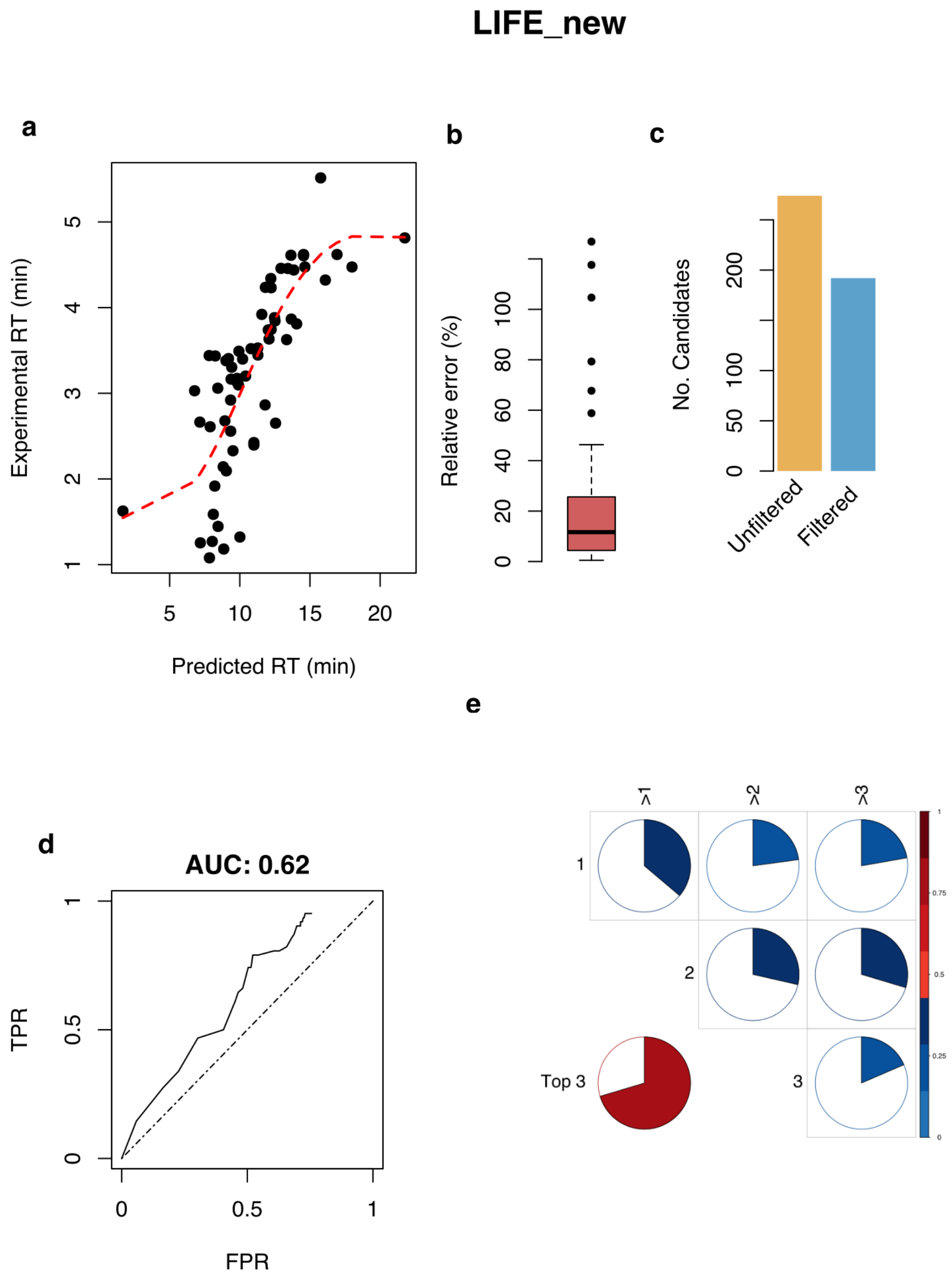

Supplementary Figure 7. Predicted-experimental projections results. Panels **a** shows the projection between the experimental and predicted RT. Panel **b** shows relative errors between the experimental and predicted RT. Box plots represent median value and their error bars represent the interquartile range (25%-75% percentiles). Panel **c** shows the number of putative candidates before and after filtering by RT error threshold. Panels **d** shows the receiver operating characteristic (ROC) curves with their respective area under curve (AUC) values. Panels **e** shows the candidate ranking. Ranking panels show the percentage of molecules in which the correct identity is exactly the first, second or third top candidate (y-axis, correct candidate ranking) and there are more than 1, 2 or 3 putative candidates in total (x-axis, total number of candidates). The *top 3* cell shows the percentage of cases in which the correct identity was ranked among the top 3 candidates of the total of cases with more than 3 candidates.

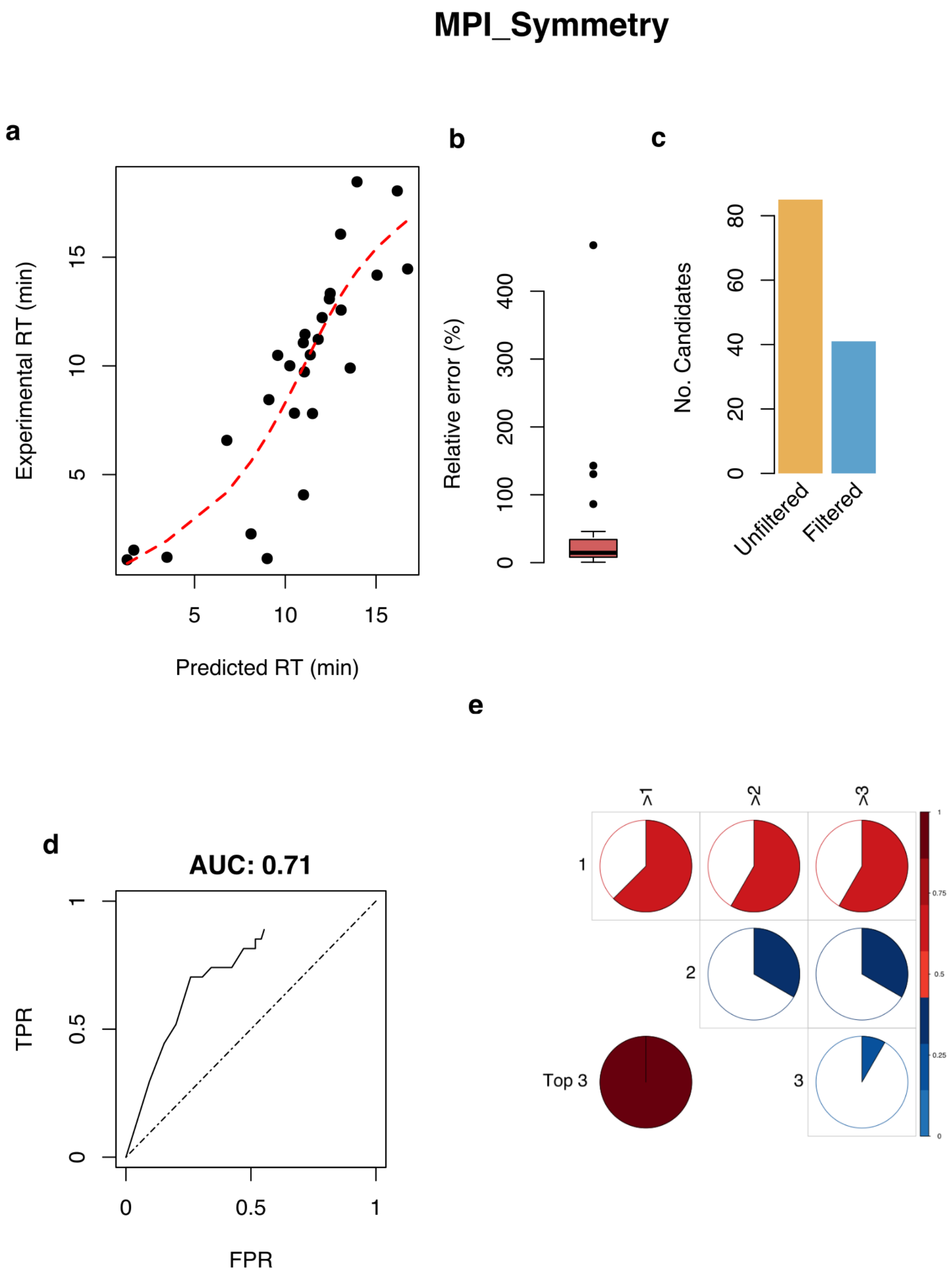

Supplementary Figure 8. Predicted-experimental projections results. Panels **a** shows the projection between the experimental and predicted RT. Panel **b** shows relative errors between the experimental and predicted RT. Box plots represent median value and their error bars represent the interquartile range (25%-75% percentiles). Panel **c** shows the number of putative candidates before and after filtering by RT error threshold. Panels **d** shows the receiver operating characteristic (ROC) curves with their respective area under curve (AUC) values. Panels **e** shows the candidate ranking. Ranking panels show the percentage of molecules in which the correct identity is exactly the first, second or third top candidate (y-axis, correct candidate ranking) and there are more than 1, 2 or 3 putative candidates in total (x-axis, total number of candidates). The *top 3* cell shows the percentage of cases in which the correct identity was ranked among the top 3 candidates of the total of cases with more than 3 candidates.

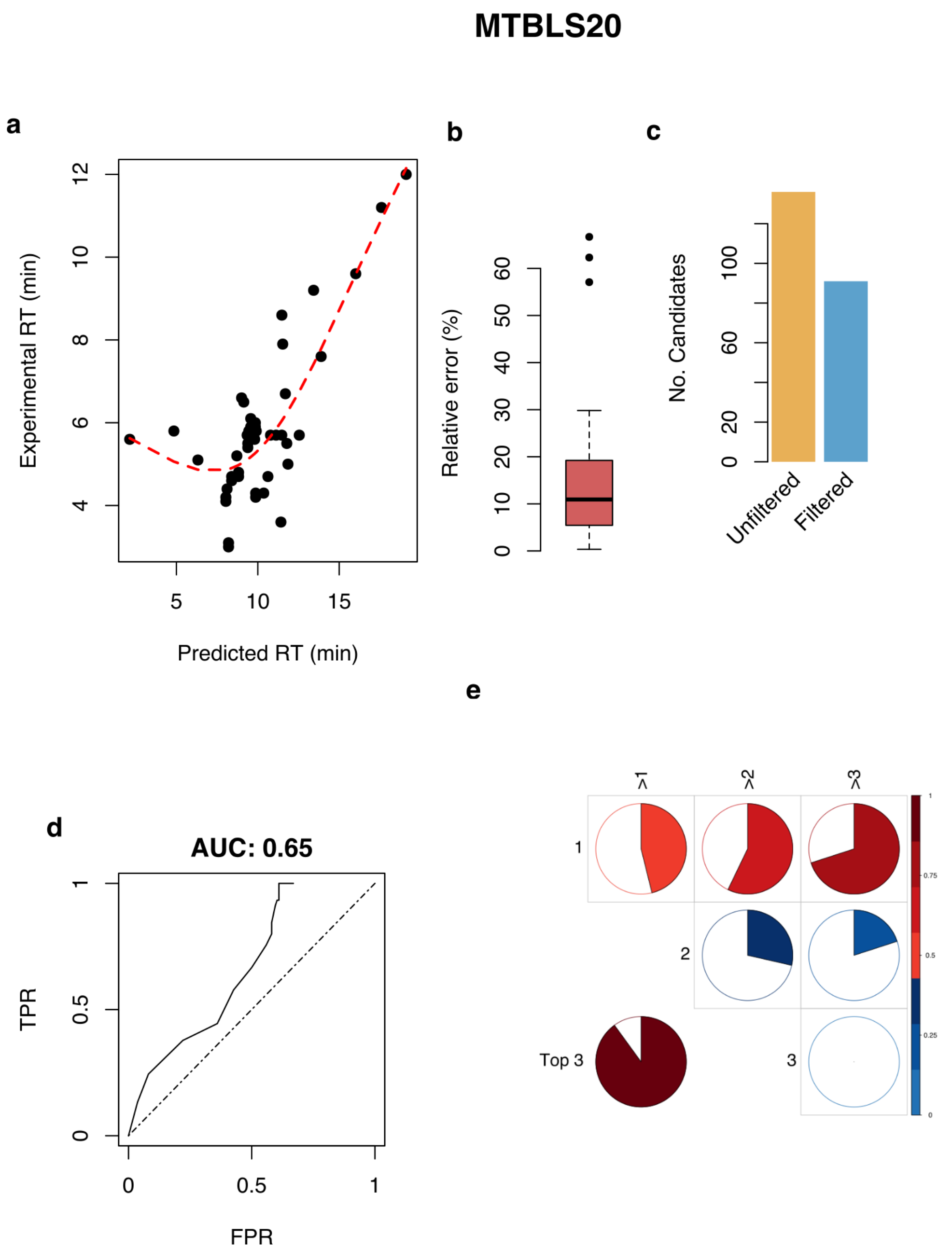

Supplementary Figure 9. Predicted-experimental projections results. Panels **a** shows the projection between the experimental and predicted RT. Panel **b** shows relative errors between the experimental and predicted RT. Box plots represent median value and their error bars represent the interquartile range (25%-75% percentiles). Panel **c** shows the number of putative candidates before and after filtering by RT error threshold. Panels **d** shows the receiver operating characteristic (ROC) curves with their respective area under curve (AUC) values. Panels **e** shows the candidate ranking. Ranking panels show the percentage of molecules in which the correct identity is exactly the first, second or third top candidate (y-axis, correct candidate ranking) and there are more than 1, 2 or 3 putative candidates in total (x-axis, total number of candidates). The *top 3* cell shows the percentage of cases in which the correct identity was ranked among the top 3 candidates of the total of cases with more than 3 candidates.

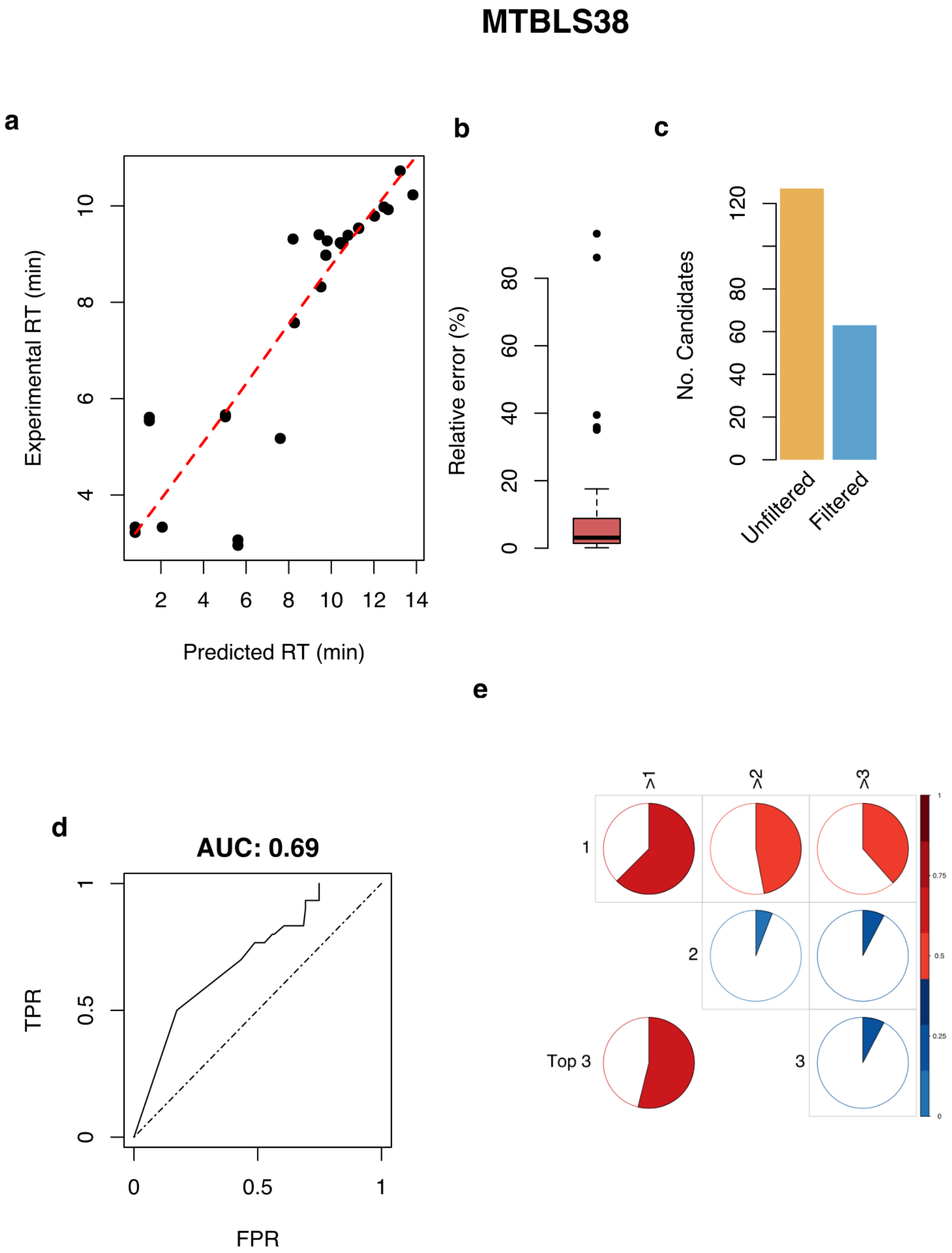

Supplementary Figure 10. Predicted-experimental projections results. Panels **a** shows the projection between the experimental and predicted RT. Panel **b** shows relative errors between the experimental and predicted RT. Box plots represent median value and their error bars represent the interquartile range (25%-75% percentiles). Panel **c** shows the number of putative candidates before and after filtering by RT error threshold. Panels **d** shows the receiver operating characteristic (ROC) curves with their respective area under curve (AUC) values. Panels **e** shows the candidate ranking. Ranking panels show the percentage of molecules in which the correct identity is exactly the first, second or third top candidate (y-axis, correct candidate ranking) and there are more than 1, 2 or 3 putative candidates in total (x-axis, total number of candidates). The *top 3* cell shows the percentage of cases in which the correct identity was ranked among the top 3 candidates of the total of cases with more than 3 candidates.

PFR-TK72

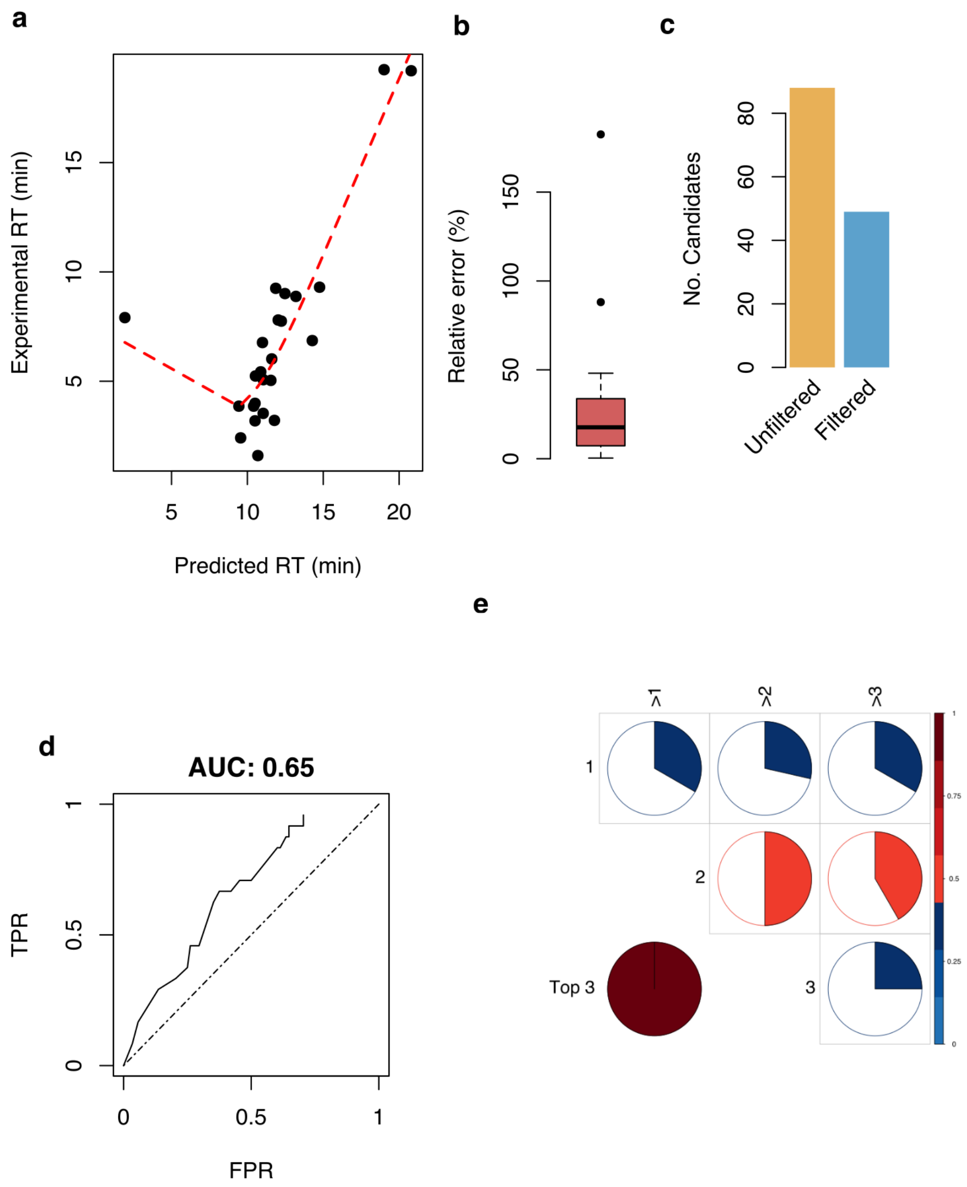

Supplementary Figure 11. Predicted-experimental projections results. Panels **a** shows the projection between the experimental and predicted RT. Panel **b** shows relative errors between the experimental and predicted RT. Box plots represent median value and their error bars represent the interquartile range (25%-75% percentiles). Panel **c** shows the number of putative candidates before and after filtering by RT error threshold. Panels **d** shows the receiver operating characteristic (ROC) curves with their respective area under curve (AUC) values. Panels **e** shows the candidate ranking. Ranking panels show the percentage of molecules in which the correct identity is exactly the first, second or third top candidate (y-axis, correct candidate ranking) and there are more than 1, 2 or 3 putative candidates in total (x-axis, total number of candidates). The *top 3* cell shows the percentage of cases in which the correct identity was ranked among the top 3 candidates of the total of cases with more than 3 candidates.

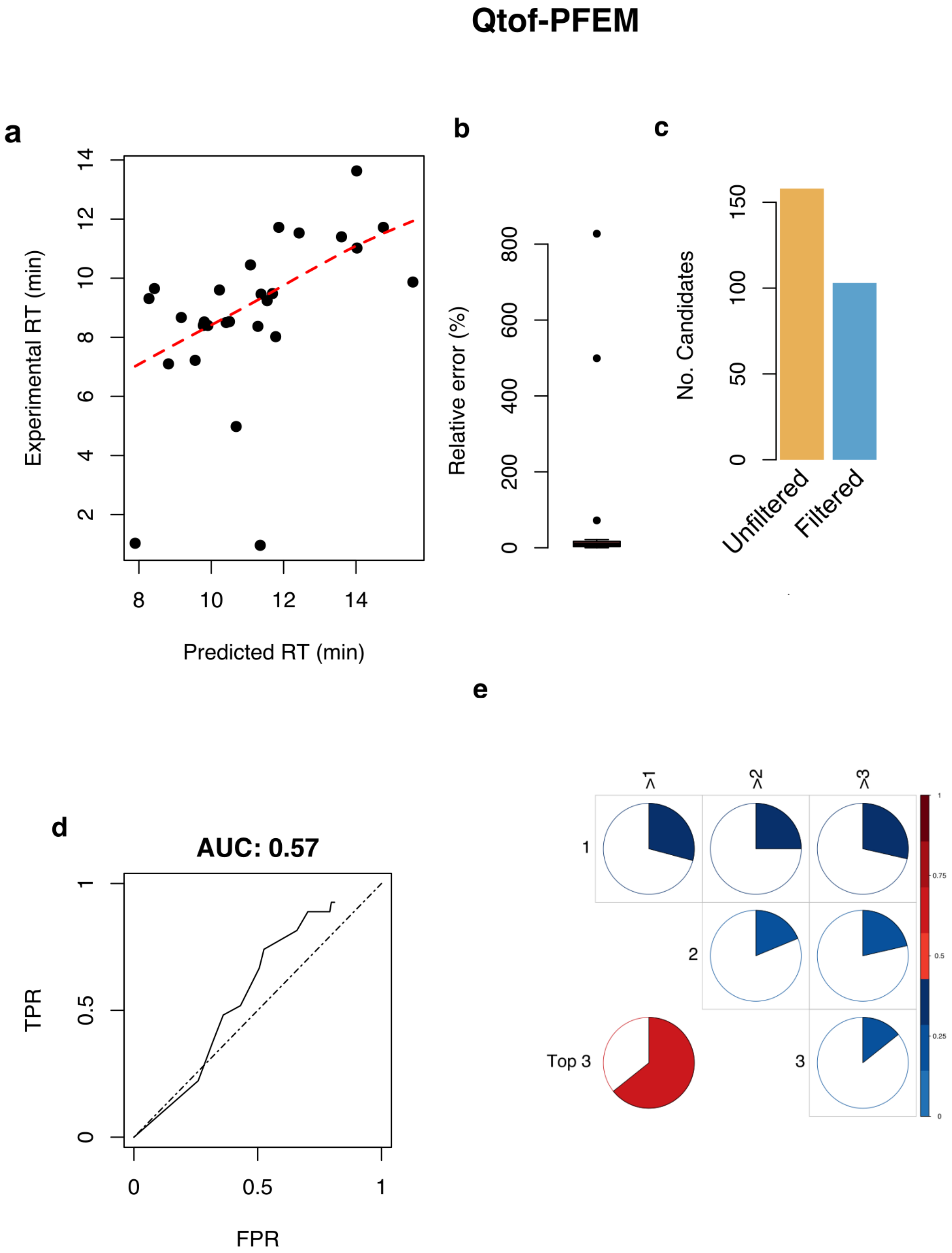

Supplementary Figure 12. Predicted-experimental projections results. Panels **a** shows the projection between the experimental and predicted RT. Panel **b** shows relative errors between the experimental and predicted RT. Box plots represent median value and their error bars represent the interquartile range (25%-75% percentiles). Panel **c** shows the number of putative candidates before and after filtering by RT error threshold. Panels **d** shows the receiver operating characteristic (ROC) curves with their respective area under curve (AUC) values. Panels **e** shows the candidate ranking. Ranking panels show the percentage of molecules in which the correct identity is exactly the first, second or third top candidate (y-axis, correct candidate ranking) and there are more than 1, 2 or 3 putative candidates in total (x-axis, total number of candidates). The *top 3* cell shows the percentage of cases in which the correct identity was ranked among the top 3 candidates of the total of cases with more than 3 candidates.

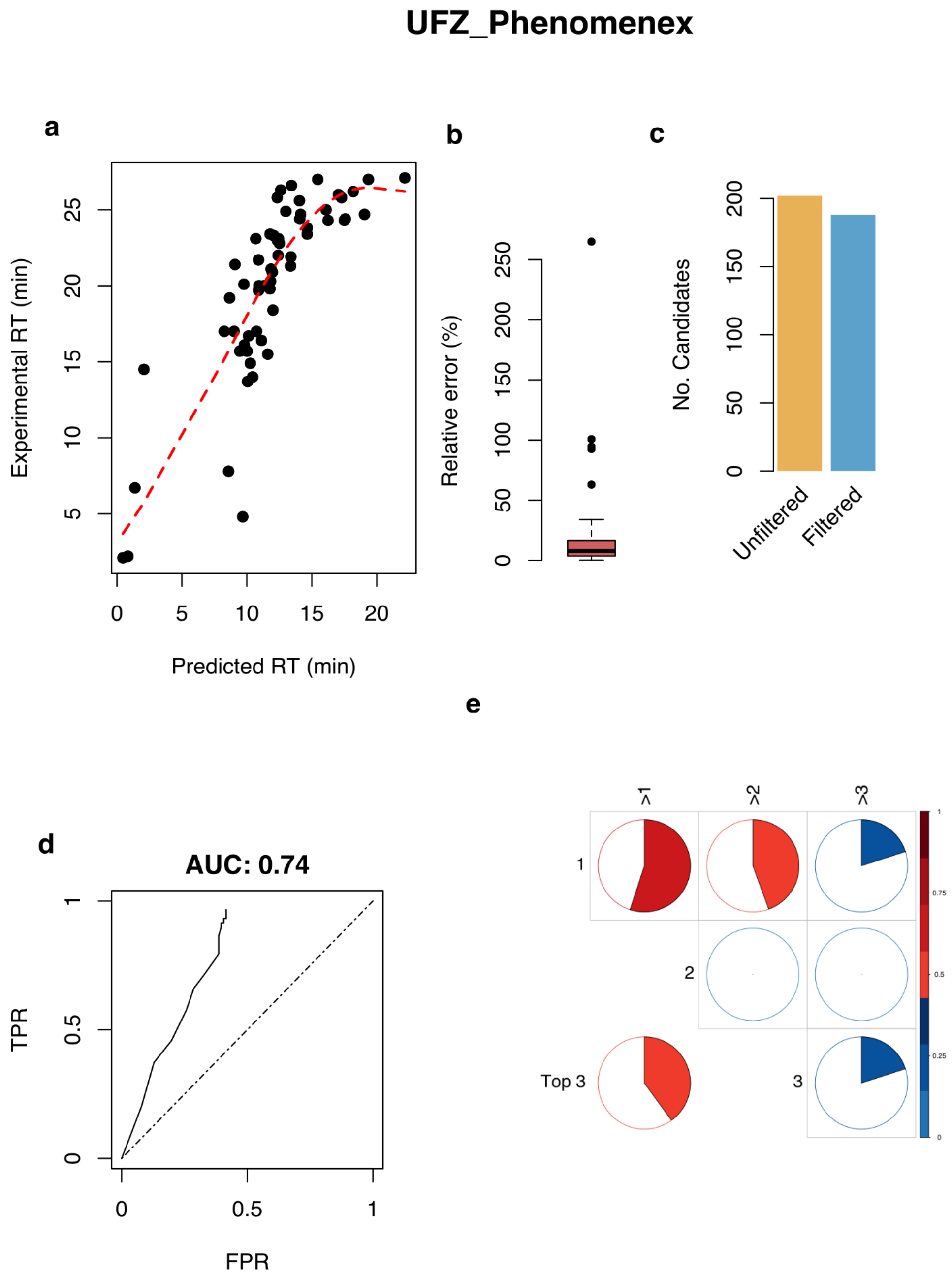

Supplementary Figure 13. Predicted-experimental projections results. Panels **a** shows the projection between the experimental and predicted RT. Panel **b** shows relative errors between the experimental and predicted RT. Box plots represent median value and their error bars represent the interquartile range (25%-75% percentiles). Panel **c** shows the number of putative candidates before and after filtering by RT error threshold. Panels **d** shows the receiver operating characteristic (ROC) curves with their respective area under curve (AUC) values. Panels **e** shows the candidate ranking. Ranking panels show the percentage of molecules in which the correct identity is exactly the first, second or third top candidate (y-axis, correct candidate ranking) and there are more than 1, 2 or 3 putative candidates in total (x-axis, total number of candidates). The *top 3* cell shows the percentage of cases in which the correct identity was ranked among the top 3 candidates of the total of cases with more than 3 candidates.

UniToyama_Atlantis

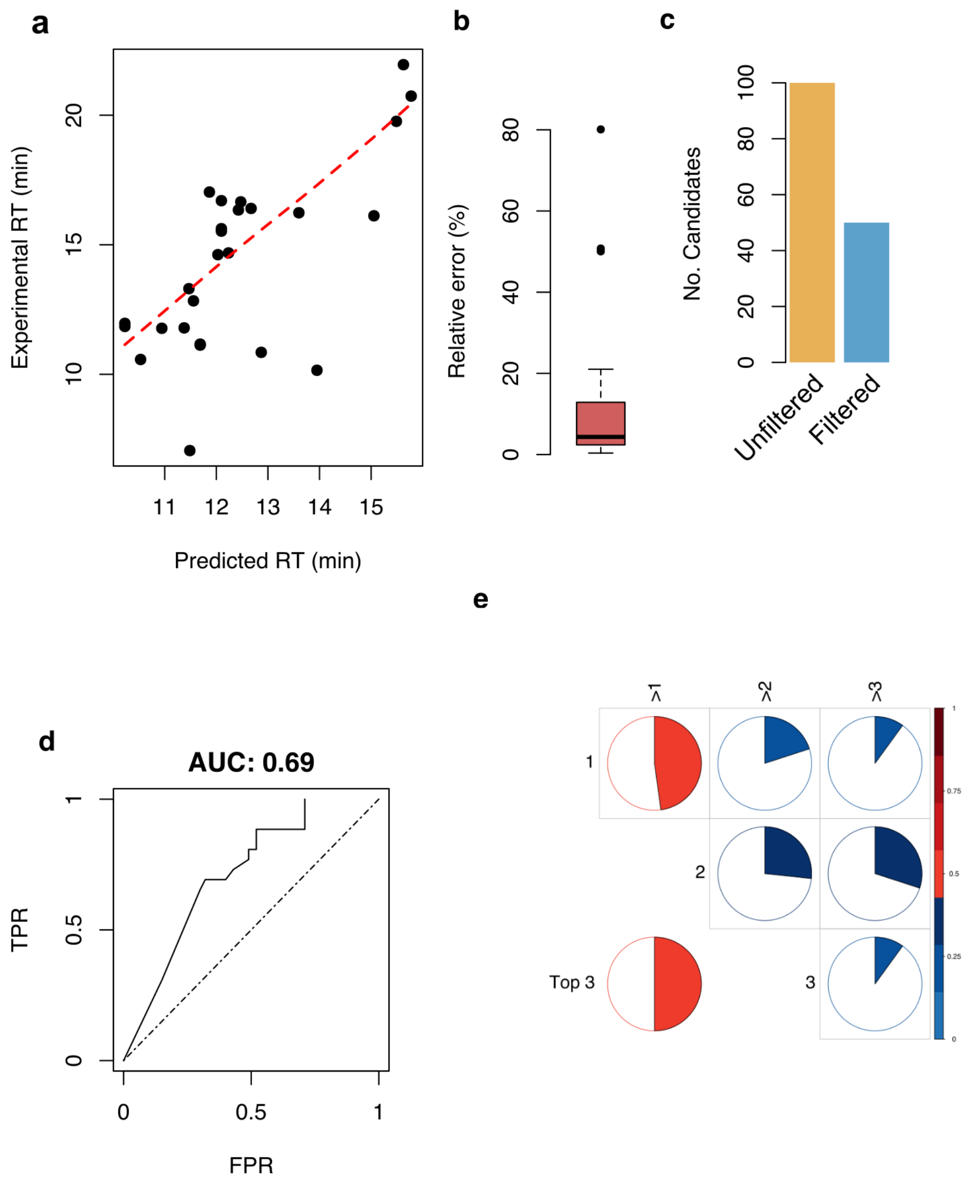

Supplementary Figure 14. Predicted-experimental projections results. Panels **a** shows the projection between the experimental and predicted RT. Panel **b** shows relative errors between the experimental and predicted RT. Box plots represent median value and their error bars represent the interquartile range (25%-75% percentiles). Panel **c** shows the number of putative candidates before and after filtering by RT error threshold. Panels **d** shows the receiver operating characteristic (ROC) curves with their respective area under curve (AUC) values. Panels **e** shows the candidate ranking. Ranking panels show the percentage of molecules in which the correct identity is exactly the first, second or third top candidate (y-axis, correct candidate ranking) and there are more than 1, 2 or 3 putative candidates in total (x-axis, total number of candidates). The *top 3* cell shows the percentage of cases in which the correct identity was ranked among the top 3 candidates of the total of cases with more than 3 candidates.

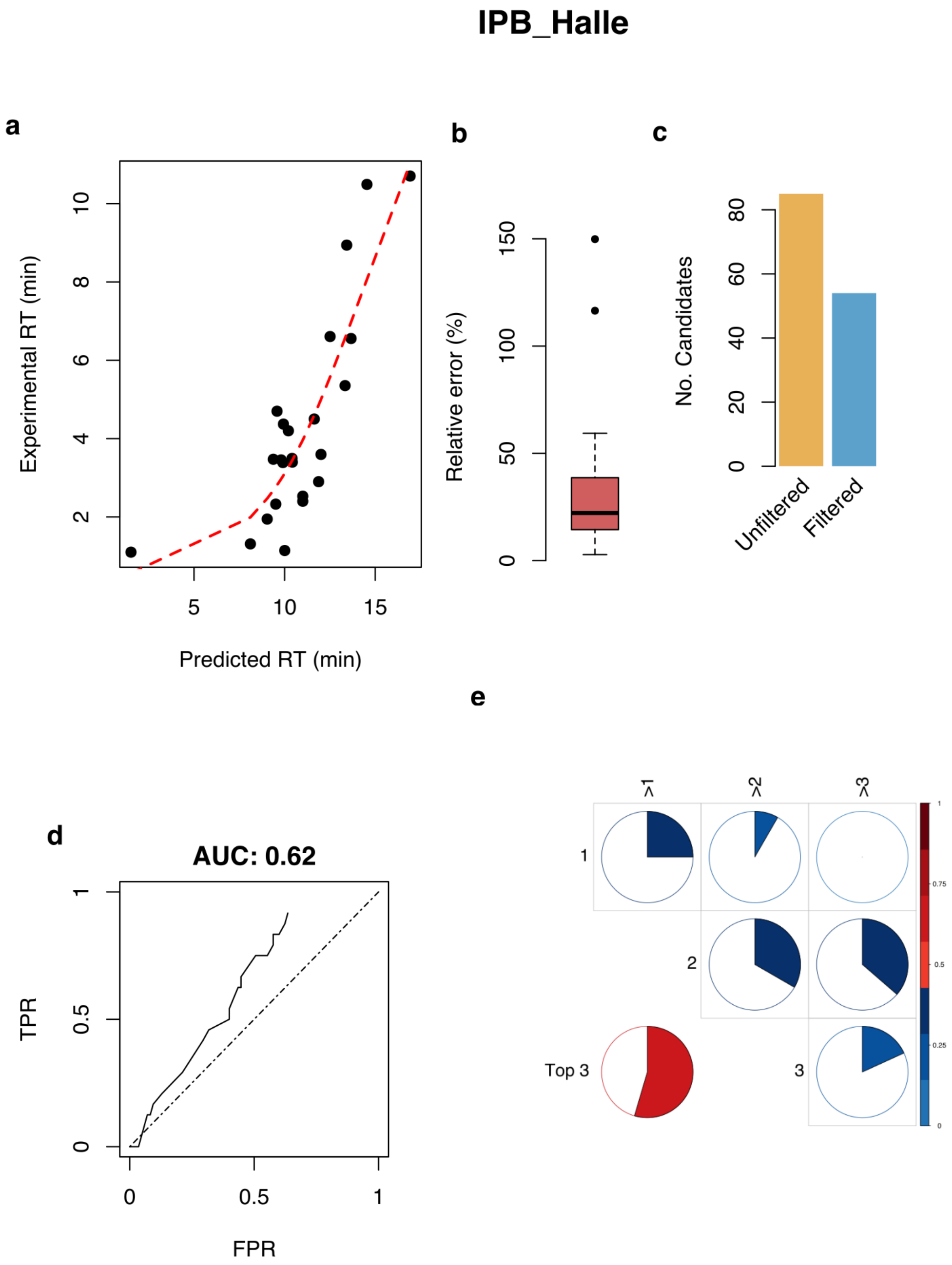

Supplementary Figure 15. Predicted-experimental projections results. Panels **a** shows the projection between the experimental and predicted RT. Panel **b** shows relative errors between the experimental and predicted RT. Box plots represent median value and their error bars represent the interquartile range (25%-75% percentiles). Panel **c** shows the number of putative candidates before and after filtering by RT error threshold. Panels **d** shows the receiver operating characteristic (ROC) curves with their respective area under curve (AUC) values. Panels **e** shows the candidate ranking. Ranking panels show the percentage of molecules in which the correct identity is exactly the first, second or third top candidate (y-axis, correct candidate ranking) and there are more than 1, 2 or 3 putative candidates in total (x-axis, total number of candidates). The *top 3* cell shows the percentage of cases in which the correct identity was ranked among the top 3 candidates of the total of cases with more than 3 candidates.

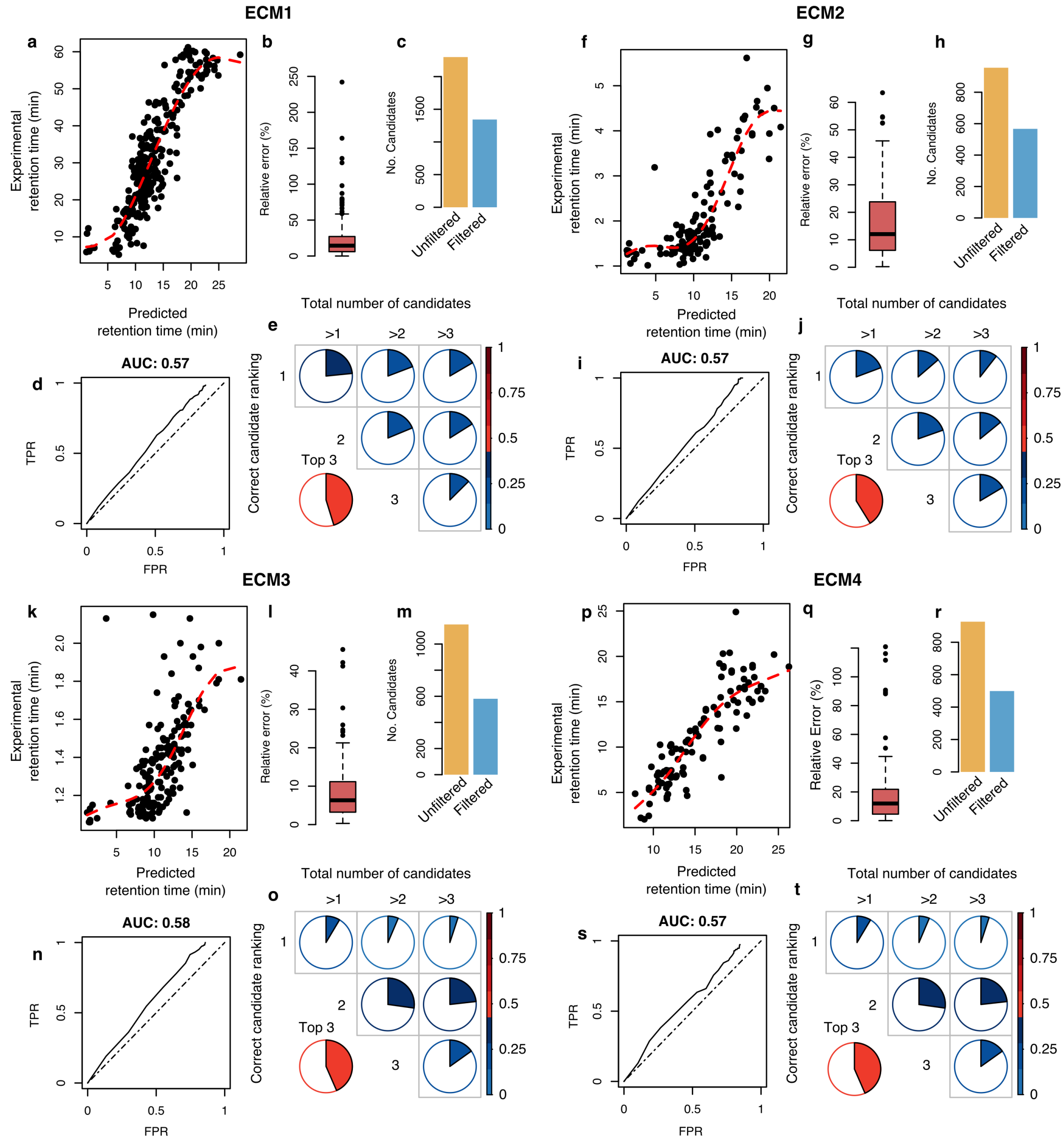

Supplementary Figure 16. Predicted-experimental projections results when using the 18,457 molecules in HMDB with PubChem number. Four chromatographic methods (CM 1 to 4) with five panels each. In the PredRet database, CM 1 to 4 are known as FEM_long, LIFE_old, RIKEN and FEM_orbitrap_plasma, respectively. Panels **a**, **f**, **k**, **p** show the projection between the experimental and predicted RT. Panels **b**, **g**, **l**, **q** show relative errors between the experimental and predicted RT. Box plots represent median value and their error bars represent the interquartile range (25%-75% percentiles). Panels **c**, **h**, **m**, **r** show the number of putative candidates before and after filtering by RT error threshold. Panels **d**, **i**, **n**, **s** show the receiver operating characteristic (ROC) curves with their respective area under curve (AUC) values. Panels **e**, **j**, **o**, **t** show the candidate ranking. Ranking panels show the percentage of molecules in which the correct identity is exactly the first, second or third top candidate (y-axis, correct candidate ranking) and there are more than 1, 2 or 3 putative candidates in total (x-axis, total number of candidates). The *top 3* cell shows the percentage of cases in which the correct identity was ranked among the top 3 candidates of the total of cases with more than 3 candidates.
